# Foreign Body Ingestion: An Unusual Case in a Patient with Dementia

**DOI:** 10.7759/cureus.41212

**Published:** 2023-06-30

**Authors:** Soham Shah, Attila Nemeth

**Affiliations:** 1 Medical School, Case Western Reserve University School of Medicine, Cleveland, USA; 2 Internal Medicine, Case Western Reserve University School of Medicine, Cleveland, USA

**Keywords:** stroke, dysphagia, geriatric emergency, dementia, foreign body ingestion

## Abstract

Foreign body ingestion is a problem seen frequently in the emergency department, particularly in children. In this case report, we present an uncommon example: foreign body ingestion in an elderly patient with a history of dementia. This patient’s symptoms of dysphagia, cough, and pooling secretions in the posterior oral cavity suggested food impaction, and after further investigation, coins were found in the upper and middle esophagus. Most of the coins were removed, the patient was monitored, and outpatient follow-up was organized to ensure the safe elimination of all the coins. This case illustrates the importance of having a high pretest probability for certain diagnoses based upon how specific patient populations present.

## Introduction

Foreign bodies, objects ingested accidentally or intentionally, have been studied in the literature for over 200 years [1,2]. They are commonly encountered in the emergency department (ED), mostly among children between 6 months and 3 years of age, and have an annual incidence of 120000 cases in the US, accounting for 1500 deaths in the United States annually [1,3]. Although encountered less frequently in adults [1], foreign body ingestion can have serious complications and therefore should always be on the differential [3].

## Case presentation

We report here a curious case of foreign body ingestion in an elderly patient. A 77-year-old man with a past medical history significant for dementia and a prior stroke with right-sided residual deficit was brought to the ED following an episode of choking and dysphagia during his breakfast 1.5 hours prior to admission.

Per the patient’s wife, he was in his normal state of health when she was giving him his breakfast. After the patient had three to four bites of food, his wife heard him gag and cough, after which he vomited once. No food products were in the vomitus. The patient then told her he couldn’t swallow and was unable to drink a glass of water. She immediately brought the patient to the ED for emergent evaluation. Upon presentation, he reported a sensation of food at the back of the throat. Otherwise, he denied chest pain, shortness of breath, or abdominal pain. He had not had any similar episodes in the past. On examination, the patient was sitting upright, anxious, and in distress, and had difficulty following commands. He was coughing, spitting out salivary secretions, and continuously trying to clear his throat. Pooling secretions were observed in the posterior oral cavity, but no obvious food particles were appreciated at the posterior pharynx. The anterior neck was not tender and we could not palpate any masses. The abdomen was soft and non-tender.

**Figure 1:**
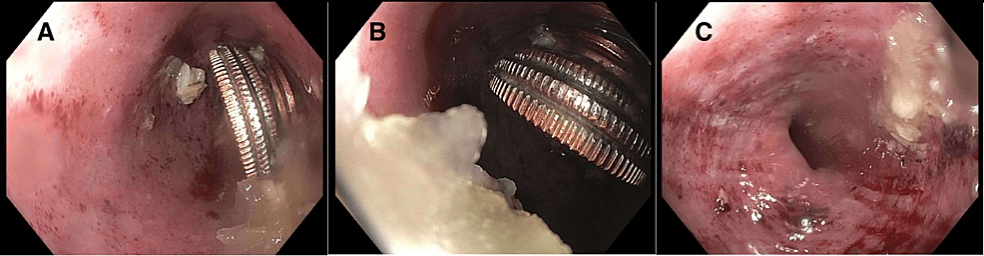
Middle third of the esophagus Figure 1A and Figure 1B: Foreign bodies in the esophagus; Figure 1C: Grade C esophagitis

Food impaction was suspected, and the gastrointestinal (GI) team was consulted immediately. The patient was given glucagon 2 mg IV to promote passage of the impacted food and was emergently intubated. An esophagogastroduodenoscopy (EGD) was then performed for likely foreign body removal. Interestingly, in addition to food, coins were found (Figure 1A, Figure 1B) along with grade C erosive esophagitis (Figure 1C) and hematin in the gastric antrum.

**Figure 2:**
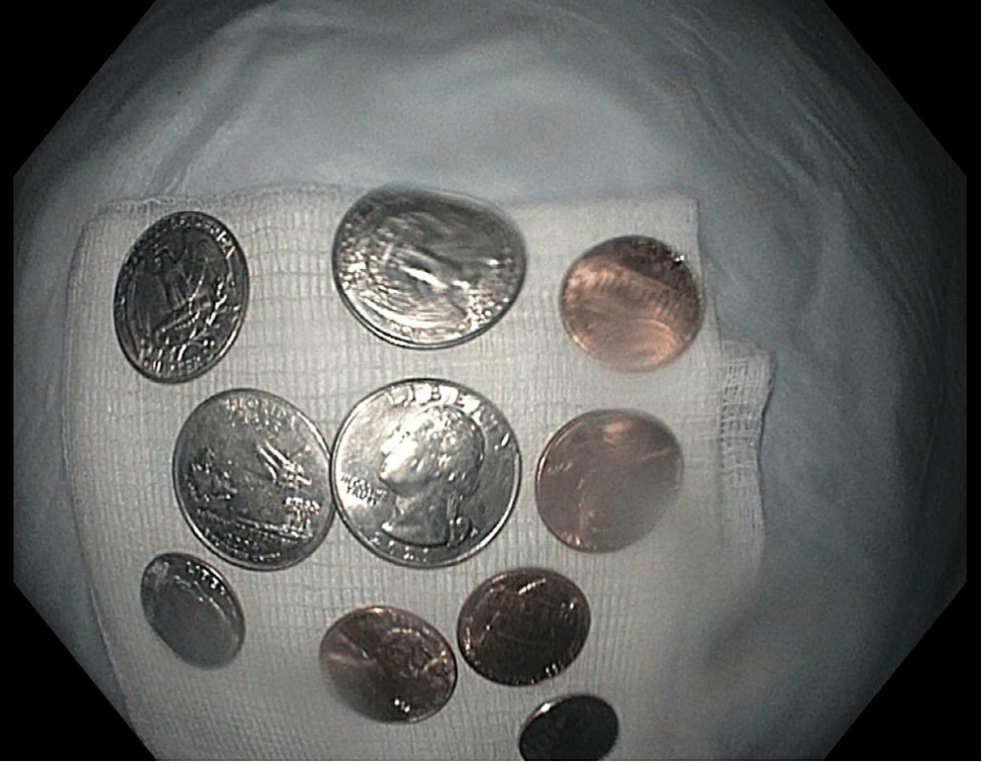
Foreign bodies (removed) Four pennies, two dimes, and four quarters ($1.24)

His wife was unaware that he swallowed the coins. Four pennies, two dimes, and four quarters ($1.24) were successfully removed from the proximal and mid-esophagus (Figure 2).

**Figure 3:**
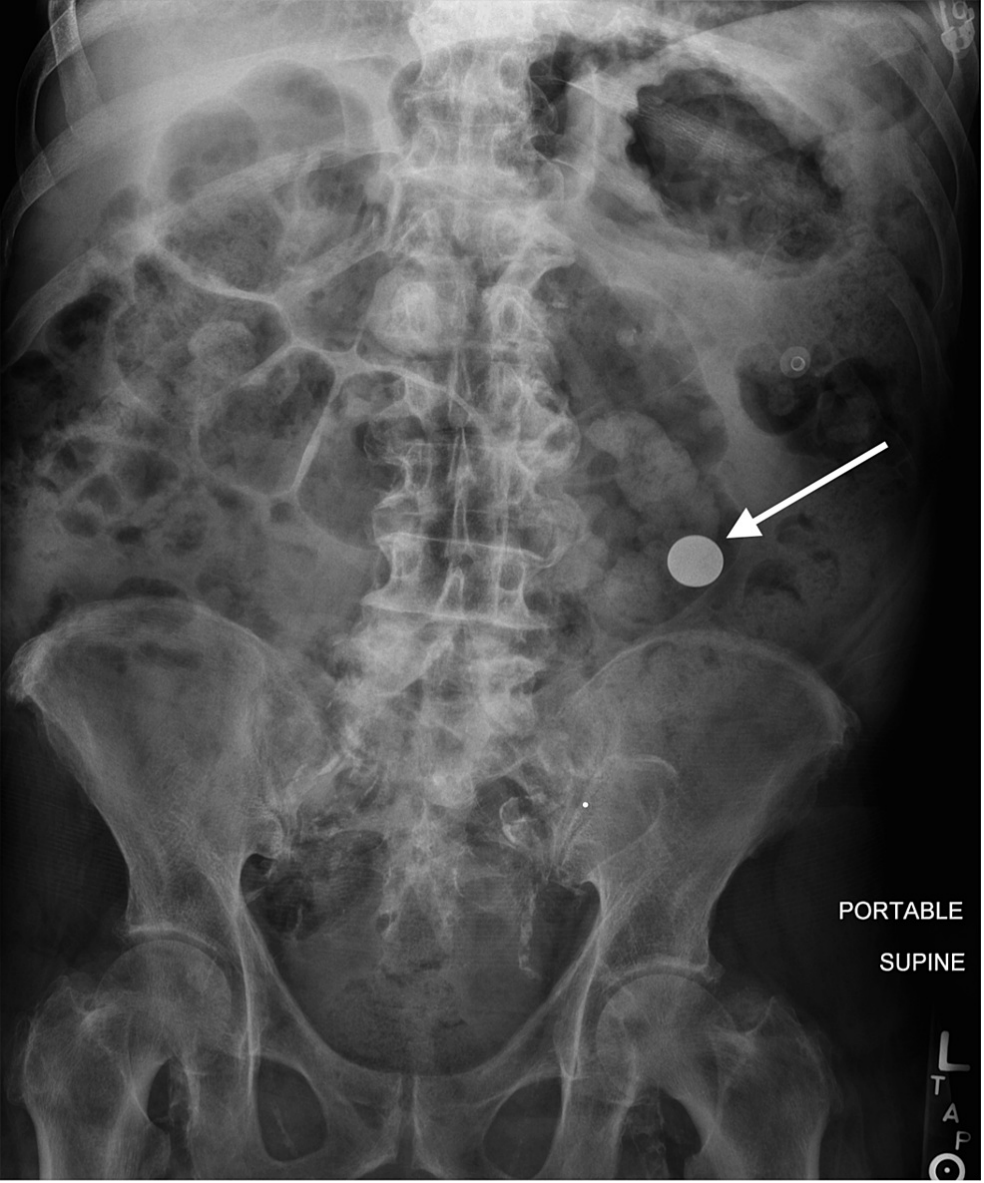
Abdominal X-ray after esophagogastroduodenoscopy (EGD)

An abdominal X-ray obtained after the EGD found one remaining coin-shaped metallic foreign body over the proximal redundant sigmoid colon (Figure 3).

The patient was admitted to medicine, serial abdominal X-rays were done, and the patient was observed for coin passage in the stool. Per GI recommendations, his diet was advanced as tolerated, starting with soft and small bite-sized food and then advancing to a regular diet. His proton pump inhibitor was continued and GI approved restarting clopidogrel. As per GI’s recommendations, a repeat abdominal X-ray was performed 7 days after discharge, along with primary care provider (PCP) follow-up, and no foreign body was identified in the colon. Also, since our team was concerned about our patient's worsening cognitive function, we recommended an updated Montreal Cognitive Assessment (MoCA) with his PCP.

## Discussion

Although many case reports discuss foreign body aspiration in children, fewer discuss cases in adults. Most cases of foreign body ingestion in adults tend to be accidental ingestion [3]. Contributory causes include acute intoxication, such as alcohol, edentulous state, cognitive slowing, and psychiatric disorders [3]. In our specific example, swallowing coins appears to have occurred as a result of his declining cognitive status related to worsening dementia. Given the patient’s history of a prior stroke, it is possible that the dementia had a vascular etiology. However, further workup is needed.

It is worth noting that cases involving foreign body ingestion can be difficult to diagnose because the patients who present to the ED are often either children or patients with psychosis or dementia [2]. As a result, it is difficult in obtaining a comprehensive history of these patient populations. Instead, the diagnosis would rely more heavily on the physical exam, presenting findings, such as sudden and complete dysphagia [2], and having a high pre-test probability for the diagnosis. Making the diagnosis quickly is paramount, as prolonged impaction was found to present a significantly increased risk for major complications [3]. In a multivariate analysis, it was shown that factors such as >12 hours lapsed between ingestion and endoscopic management, and greater sharpness of the foreign body were associated with significantly greater rates of complications [3]. The study found that other risk factors, such as gender, location in the esophagus, and presence of an esophageal stricture were not statistically significant [3].

## Conclusions

This case illustrates the importance of having a high pretest probability for certain diagnoses based upon how specific patient populations present. This is especially important when delaying or missing a diagnosis has delirious consequences for our patients, such as our case of foreign body ingestions.
